# Supplementary figures and images for: Uncovering the effects of model initialization on deep model generalization: A study with adult and pediatric chest X-ray images
Source: PLOS Digit Health. 2024 Jan 17;3(1):e0000286. doi: 10.1371/journal.pdig.0000286 (PMC10793885; doi:10.1371/journal.pdig.0000286)

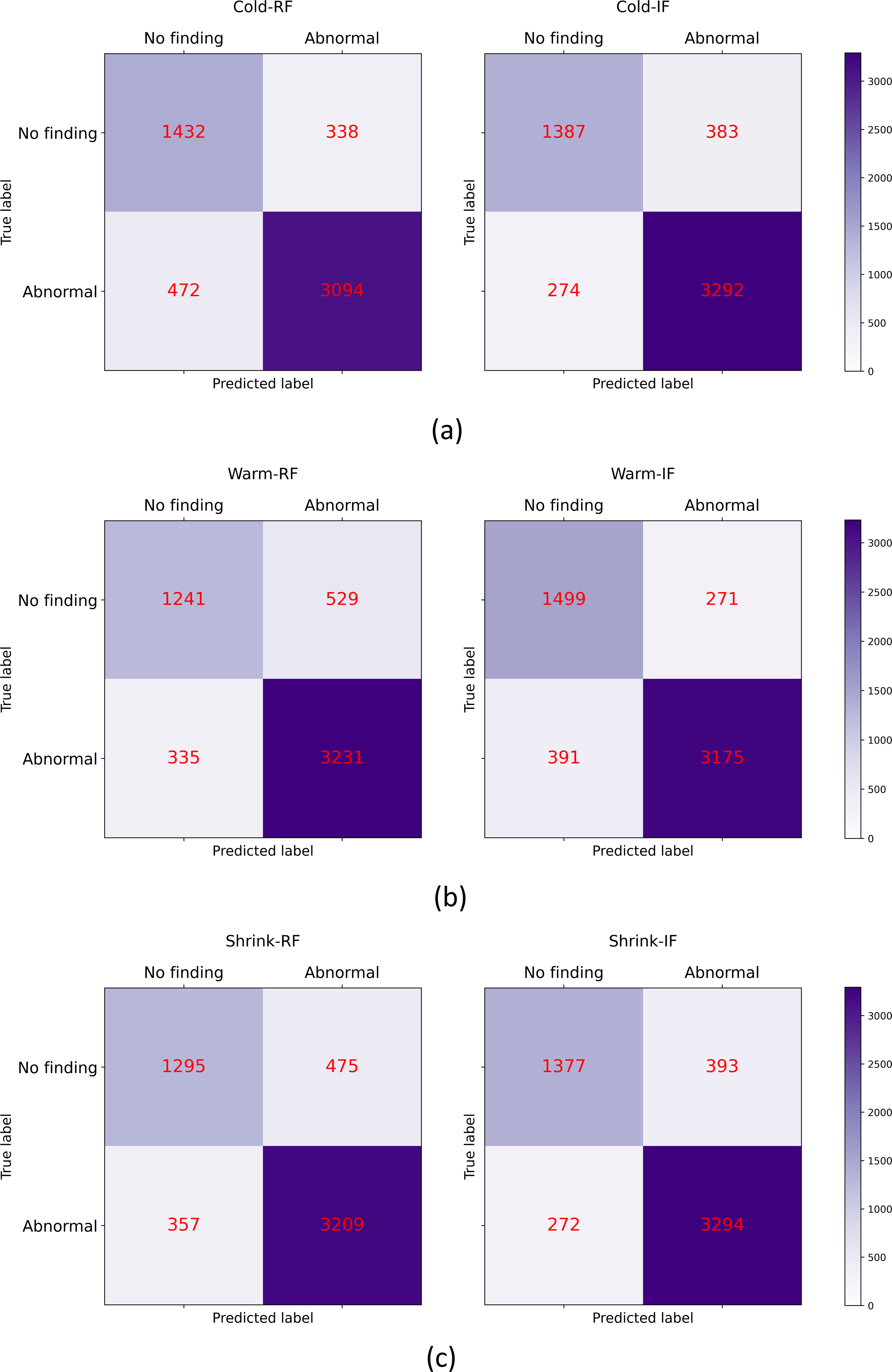

Supplement: S1 Fig — (TIF) [file pdig.0000286.s001.tif]

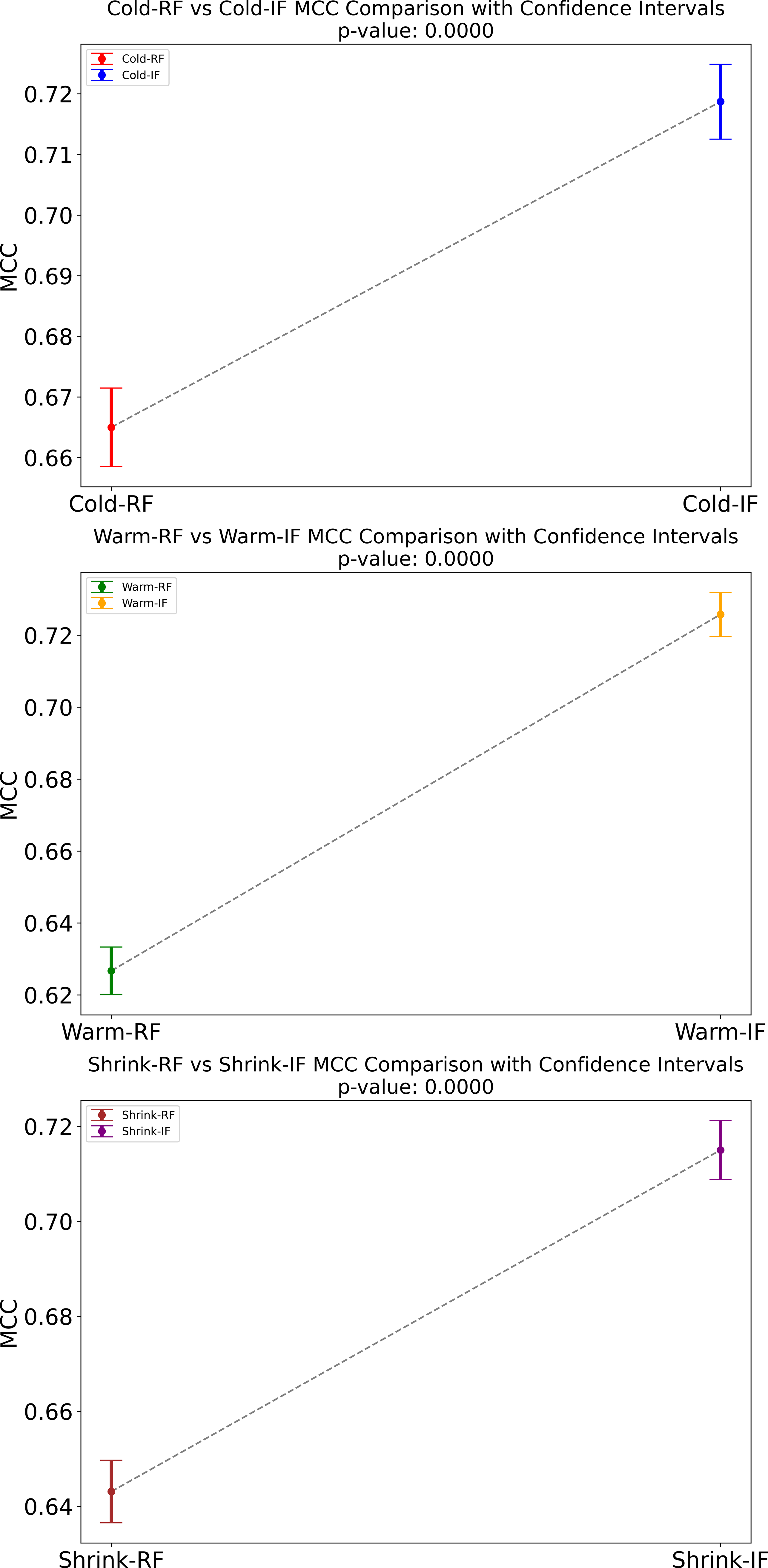

Supplement: S2 Fig — (TIF) [file pdig.0000286.s002.tif]

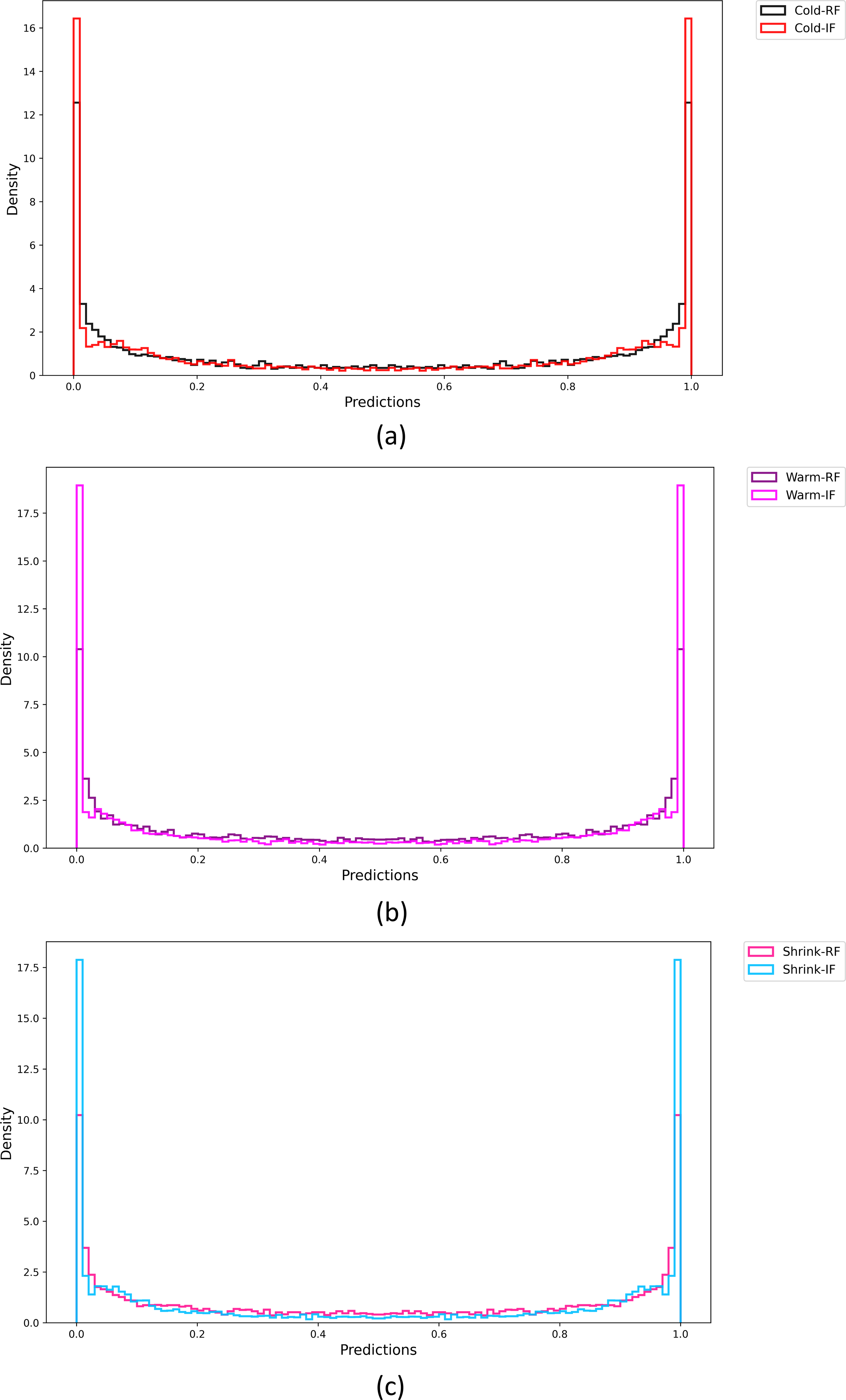

Supplement: S3 Fig — (a) Cold-RF and Cold-IF; (b) Warm-RF and Warm-IF, and (c) Shrink-RF and Shrink-IF. (TIF) [file pdig.0000286.s003.tif]
