# Supplementary material for: Uncovering the effects of model initialization on deep model generalization: A study with adult and pediatric chest X-ray images
Source: PLOS Digit Health. 2024 Jan 17;3(1):e0000286. doi: 10.1371/journal.pdig.0000286 (PMC10793885; doi:10.1371/journal.pdig.0000286)
Supplement: S1 Table — Bold numerical values denote superior performance in their respective columns. The * denotes statistically significant precision (p<0.00001) compared to the baseline. (DOCX) [file pdig.0000286.s004.docx]

**S1 Table.** **Performances achieved with the external adult test.** Bold numerical values denote superior performance in their respective columns. The * denotes statistically significant precision (*p*<0.00001) compared to the baseline.

| **Models** | **AUPRC** | **B. Acc.** | **P** | **R** | **F** | **MCC** |
| --- | --- | --- | --- | --- | --- | --- |
| Cold-IF-Baseline | 0.8490 | 0.7170 | 0.8452 | 0.5807 | 0.6884 | 0.4378 (0.4226,0.4530) |
| **EWA Ensemble** | | | | | | |
| Cold-IF, Warm-IF | 0.8557 | **0.7272** | 0.8519 | **0.5976** | **0.7024** | **0.4568 (0.4415,0.4721)** |
| Cold-IF, Shrink-IF | 0.8485 | 0.7146 | 0.8575 | 0.5568 | 0.6752 | 0.4375 (0.4223,0.4527) |
| Warm-IF, Shrink-IF | 0.8522 | 0.6932 | 0.8802 | 0.4756 | 0.6175 | 0.4113 (0.3962,0.4264) |
| Cold-IF, Warm-IF, Shrink-IF | 0.8515 | 0.722 | 0.8454 | 0.5934 | 0.6973 | 0.4461 (0.4308,0.4614) |
| **F-SLSQP Ensemble** | | | | | | |
| Cold-IF, Warm-IF | **0.8591** | 0.6961 | **0.8929*** | 0.4697 | 0.6156 | 0.4205 (0.4053,0.4357) |
| Cold-IF, Shrink-IF | 0.8500 | 0.7013 | 0.8762 | 0.5000 | 0.6367 | 0.4225 (0.4073,0.4377) |
| Warm-IF, Shrink-IF | 0.8509 | 0.7032 | 0.8689 | 0.5130 | 0.6451 | 0.4229 (0.4077,0.4381) |
| Cold-IF, Warm-IF, Shrink-IF | 0.8520 | 0.7144 | 0.8626 | 0.5492 | 0.6711 | 0.4386 (0.4234,0.4538) |
| **AGELFS** | | | | | | |
| Cold-IF, Warm-IF | 0.8521 | 0.6878 | 0.8846 | 0.4579 | 0.6034 | 0.4047 (0.3896,0.4198) |
| Cold-IF, Shrink-IF | 0.8474 | 0.7002 | 0.8618 | 0.5139 | 0.6439 | 0.4156 (0.4005,0.4307) |
| Warm-IF, Shrink-IF | 0.8492 | 0.7032 | 0.8641 | 0.5189 | 0.6484 | 0.4213 (0.4061,0.4365) |
| Cold-IF, Warm-IF, Shrink-IF | 0.8494 | 0.7064 | 0.8609 | 0.5311 | 0.6569 | 0.4253 (0.4101,0.4405) |
