# Supplementary material for: Uncovering the effects of model initialization on deep model generalization: A study with adult and pediatric chest X-ray images
Source: PLOS Digit Health. 2024 Jan 17;3(1):e0000286. doi: 10.1371/journal.pdig.0000286 (PMC10793885; doi:10.1371/journal.pdig.0000286)
Supplement: S2 Table — Bold numerical values denote superior performance in their respective columns. The * denotes statistically significant recall (p<0.00001) compared to the baseline. (DOCX) [file pdig.0000286.s005.docx]

**S2 Table. Performances achieved with the external Ped-2 test.** Bold numerical values denote superior performance in their respective columns. The * denotes statistically significant recall (*p*<0.00001) compared to the baseline.

| Models | AUPRC | B. Acc. | P | R | F | MCC |
| --- | --- | --- | --- | --- | --- | --- |
| Cold-IF-Baseline | **0.4685** | **0.5480** | 0.3997 | 0.8794 | **0.5496** | **0.1206 (0.1118,0.1294)** |
| EWA Ensemble | | | | | | |
| Cold-IF, Warm-IF | 0.4388 | 0.5278 | 0.3869 | 0.9280 | 0.5461 | 0.0869 (0.0793,0.0945) |
| Cold-IF, Shrink-IF | 0.4476 | 0.5333 | 0.3902 | 0.9105 | 0.5463 | 0.0952 (0.0873,0.1031) |
| Warm-IF, Shrink-IF | 0.4375 | 0.5368 | 0.3934 | 0.8644 | 0.5407 | 0.0923 (0.0845,0.1001) |
| Cold-IF, Warm-IF, Shrink-IF | 0.4367 | 0.5240 | 0.3847 | **0.9335*** | 0.5449 | 0.0785 (0.0713,0.0857) |
| F-SLSQP Ensemble | | | | | | |
| Cold-IF, Warm-IF | 0.4294 | 0.5379 | 0.3942 | 0.8589 | 0.5404 | 0.0937 (0.0859,0.1015) |
| Cold-IF, Shrink-IF | 0.4550 | 0.5379 | 0.3942 | 0.8564 | 0.5399 | 0.0931 (0.0853,0.1009) |
| Warm-IF, Shrink-IF | 0.4494 | 0.5370 | 0.3931 | 0.8794 | 0.5433 | 0.0960 (0.0881,0.1039) |
| Cold-IF, Warm-IF, Shrink-IF | 0.4420 | 0.5308 | 0.3889 | 0.9070 | 0.5444 | 0.0880 (0.0804,0.0956) |
| AGELFS | | | | | | |
| Cold-IF, Warm-IF | 0.4309 | 0.5456 | 0.3997 | 0.8354 | 0.5407 | 0.1061 (0.0978,0.1144) |
| Cold-IF, Shrink-IF | 0.4567 | 0.5475 | **0.4003** | 0.8529 | 0.5449 | 0.1135 (0.1050,0.1220) |
| Warm-IF, Shrink-IF | 0.4255 | 0.5416 | 0.3960 | 0.8724 | 0.5447 | 0.1046 (0.0082,0.1128) |
| Cold-IF, Warm-IF, Shrink-IF | 0.4255 | 0.5389 | 0.3943 | 0.8784 | 0.5443 | 0.1001 (0.0920,0.1082) |
