# Supplementary material for: Uncovering the effects of model initialization on deep model generalization: A study with adult and pediatric chest X-ray images
Source: PLOS Digit Health. 2024 Jan 17;3(1):e0000286. doi: 10.1371/journal.pdig.0000286 (PMC10793885; doi:10.1371/journal.pdig.0000286)
Supplement: S3 Table — Bold numerical values denote superior performance in their respective columns. The * denotes statistically significant recall (p<0.00001) compared to the baseline. (DOCX) [file pdig.0000286.s006.docx]

**S3 Table. Performances achieved with the external Ped-11 test.** Bold numerical values denote superior performance in their respective columns. The * denotes statistically significant recall (*p*<0.00001) compared to the baseline.

| Models | AUPRC | B. Acc. | P | R | F | MCC |
| --- | --- | --- | --- | --- | --- | --- |
| Warm-IF-Baseline | 0.5381 | **0.6446** | 0.4368 | 0.6976 | 0.5372 | 0.2681 (0.2559,0.2803) |
| EWA Ensemble | | | | | | |
| Cold-IF, Warm-IF | 0.5381 | 0.6222 | 0.3963 | 0.7918 | 0.5282 | 0.2336 (0.2220,0.2452) |
| Cold-IF, Shrink-IF | 0.5391 | 0.6308 | 0.4103 | 0.7519 | 0.5309 | 0.2449 (0.2331,0.2567) |
| Warm-IF, Shrink-IF | 0.5422 | 0.6407 | 0.4286 | 0.7126 | 0.5353 | 0.2609 (0.2488,0.2730) |
| Cold-IF, Warm-IF, Shrink-IF | 0.5380 | 0.6180 | 0.3924 | **0.7943*** | 0.5253 | 0.2267 (0.2152,0.2382) |
| F-SLSQP Ensemble | | | | | | |
| Cold-IF, Warm-IF | 0.5384 | 0.6414 | 0.4319 | 0.7020 | 0.5348 | 0.2622 (0.2501,0.2743) |
| Cold-IF, Shrink-IF | 0.5474 | 0.6442 | 0.4346 | 0.7045 | 0.5376 | 0.2674 (0.2552,0.2796) |
| Warm-IF, Shrink-IF | 0.5462 | 0.6337 | 0.4179 | 0.7269 | 0.5307 | 0.2487 (0.2368,0.2606) |
| Cold-IF, Warm-IF, Shrink-IF | 0.5418 | 0.6274 | 0.4061 | 0.7575 | 0.5287 | 0.2393 (0.2276,0.2510) |
| AGELFS | | | | | | |
| Cold-IF, Warm-IF | 0.5450 | 0.6422 | **0.4374** | 0.6833 | 0.5334 | 0.2636 (0.2515,0.2757) |
| Cold-IF, Shrink-IF | **0.5528** | 0.6420 | 0.4297 | 0.7145 | 0.5367 | 0.2635 (0.2514,0.2756) |
| Warm-IF, Shrink-IF | 0.5407 | 0.6383 | 0.4232 | 0.7251 | 0.5345 | 0.2569 (0.2449,0.2689) |
| Cold-IF, Warm-IF, Shrink-IF | 0.5411 | 0.6414 | 0.4236 | 0.7394 | **0.5386** | 0.2631 (0.2510,0.2752) |
