# Supplementary material for: Uncovering the effects of model initialization on deep model generalization: A study with adult and pediatric chest X-ray images
Source: PLOS Digit Health. 2024 Jan 17;3(1):e0000286. doi: 10.1371/journal.pdig.0000286 (PMC10793885; doi:10.1371/journal.pdig.0000286)
Supplement: S4 Table — Bold numerical values denote superior performance in their respective columns. The * denotes statistically significant recall (p<0.00007) compared to the baseline. (DOCX) [file pdig.0000286.s007.docx]

**S4 Table. Performances achieved with the external Ped-18 test.** Bold numerical values denote superior performance in their respective columns. The * denotes statistically significant recall (*p*<0.00007) compared to the baseline.

| Models | AUPRC | B. Acc. | P | R | F | MCC |
| --- | --- | --- | --- | --- | --- | --- |
| Warm-IF-Baseline | 0.6820 | 0.7324 | 0.6229 | 0.8241 | 0.7095 | 0.4614 (0.4448,0.4780) |
| EWA Ensemble | | | | | | |
| Cold-IF, Warm-IF | 0.6704 | 0.7130 | 0.5883 | 0.8582 | 0.6981 | 0.4309 (0.4145,0.4473) |
| Cold-IF, Shrink-IF | 0.6658 | 0.7192 | 0.5967 | 0.8541 | 0.7026 | 0.4414 (0.4249,0.4579) |
| Warm-IF, Shrink-IF | 0.6774 | 0.7328 | 0.6186 | 0.8371 | 0.7115 | 0.4635 (0.4469,0.4801) |
| Cold-IF, Warm-IF, Shrink-IF | 0.6698 | 0.7144 | 0.5890 | **0.8616*** | 0.6997 | 0.4342 (0.4177,0.4507) |
| F-SLSQP Ensemble | | | | | | |
| Cold-IF, Warm-IF | 0.6806 | 0.7310 | 0.6203 | 0.8262 | 0.7086 | 0.4590 (0.4425,0.4755) |
| Cold-IF, Shrink-IF | 0.6788 | 0.7214 | 0.5999 | 0.8514 | 0.7039 | 0.4447 (0.4282,0.4612) |
| Warm-IF, Shrink-IF | 0.6791 | 0.7174 | 0.5959 | 0.8494 | 0.7004 | 0.4372 (0.4207,0.4537) |
| Cold-IF, Warm-IF, Shrink-IF | 0.6722 | 0.7192 | 0.5968 | 0.8534 | 0.7024 | 0.4411 (0.4246,0.4576) |
| AGELFS | | | | | | |
| Cold-IF, Warm-IF | 0.6804 | **0.7368** | **0.6237** | 0.8371 | **0.7148** | **0.4708 (0.4542,0.4874)** |
| Cold-IF, Shrink-IF | 0.6762 | 0.7161 | 0.5925 | 0.8562 | 0.7003 | 0.4361 (0.4196,0.4526) |
| Warm-IF, Shrink-IF | 0.6849 | 0.7190 | 0.5964 | 0.8541 | 0.7024 | 0.4410 (0.4245,0.4575) |
| Cold-IF, Warm-IF, Shrink-IF | **0.6852** | 0.7178 | 0.5939 | 0.8582 | 0.7020 | 0.4397 (0.4232,0.4562) |
