# Supplementary material for: Uncovering the effects of model initialization on deep model generalization: A study with adult and pediatric chest X-ray images
Source: PLOS Digit Health. 2024 Jan 17;3(1):e0000286. doi: 10.1371/journal.pdig.0000286 (PMC10793885; doi:10.1371/journal.pdig.0000286)
Supplement: Attachment — Submitted filename: Response to Reviewers.docx [file pdig.0000286.s008.docx]

**Response to the Editor:**

Editor comments: The paper gets positive feedbacks from two expert reviewers. There are some mixed major and minor concerns, but regarding the overall impact and the addressability of the concerns, the manuscript lies in the border of minor to major revision, with more in the minor line. It would still be great if authors can address all the raised questions.

Author response: We render our sincere thanks to the Academic Editor for arranging the peer review and encouraging the resubmission of our manuscript. To the best of our knowledge and belief, we have addressed the reviewers’ concerns in the revised manuscript.

Q1: Please review your reference list to ensure that it is complete and correct. If you have cited papers that have been retracted, please include the rationale for doing so in the manuscript text, or remove these references and replace them with relevant current references. Any changes to the reference list should be mentioned in the rebuttal letter that accompanies your revised manuscript. If you need to cite a retracted article, indicate the article's retracted status in the References list and also include a citation and full reference for the retraction notice.

Author response: We confirm that the reference list is complete and correct and that we have not cited any retracted papers.

Q2: Please ensure that the funding information and financial disclosure Statement are matched. In the Funding Information, you indicated that no funding was received. Please revise the Funding information field to reflect the funding received.

Author response: Agreed. We confirm that the Funding information and Financial Disclosure Statement are matched in the revised submission. We revised the funding information field as follows: “This study is supported by the Intramural Research Program (IRP) of the National Library of Medicine (NLM) and the National Institutes of Health (NIH).”

What this means is that our research group is internally funded at the NLM/NIH through its IRP. Therefore, we do not have a grant number to provide since that indicates external funding.

Q3: Please provide separate figure files in tif or .eps format only and remove any figures embedded in your manuscript file. Please also ensure that all files are under our size limit of 10MB. For more information about figure files please see our guidelines.

Author response: We confirm that we have removed all the figures embedded in the manuscript file. The figures are separately provided in TIF format. We ensured that the size of the figures, including the striking diagram, did not exceed the recommended limit of 10MB. All figures are checked and converted using the PACE tool recommended by PLOS.

**Response to Reviewer #1:**

Q1: The authors' approach to exploring the impact of various weight initialization methods in deep learning models on the classification of chest X-rays is indeed compelling, especially with the focus on the generalizability of a model trained on adult data to the pediatric population. The manuscript presents a good analysis with both empirical and statistical analysis, though a few points would benefit from further clarification:

The author(s) have Could the authors share the factors that influenced their decision to focus on adult and pediatric CXRs for this study?

Author response: We greatly appreciate the reviewer's time and the constructive comments that have been offered, particularly recognizing the significance of our focus on the generalizability of DL models across adult and pediatric chest X-rays (CXRs). Our choice to study both adult and pediatric CXRs was motivated by several key factors that highlight the unique challenges and opportunities in this domain:

**Data Availability:** A majority of publicly available CXR datasets are focused on the adult population. This skew in data availability naturally drives the need to explore how well models trained on these adult-centered datasets generalize to pediatric cases.

**Anatomical Variability:** Pediatric and adult lungs exhibit both similarities and unique distinctions due to developmental stages. For example, pediatric patients possess thinner chest walls and more compliant rib cages, which are likely to influence the model's performance.

**Disease Presentation:** Diseases manifest differently across age groups. A “standard” presentation in adults is typically due to a strong immune response. However, children who have a developing immune system often exhibit several subtle radiological signs as disease indicators.

**Imaging Constraints:** Pediatric CXRs often follow age-specific imaging protocols that result in variations in image intensity and contrast. Furthermore, the depth of inspiration may not be consistent across patients which introduces another layer of complexity.

Our research aims to address the current gap in the literature by rigorously evaluating how different model initialization strategies influence the model's ability to generalize from adult to pediatric populations, particularly across various pediatric age groups.

Q2: The authors indicate that the training and validation sets from the RSNA dataset were divided into two equal-sized subsets to facilitate the warm-start scenario. Could the authors share any supporting references that guided this split?

Author response: We appreciate the reviewer's inquiry into the methodological details concerning our data partitioning strategy for the warm-start scenario. Our decision to divide the training and validation sets from the RSNA dataset into two equal-sized subsets was influenced by the work titled “On warm starting neural network training” [10]. In this study, the authors adopted a two-phase training approach, that is, initially training the model to convergence using one-half of the dataset and subsequently expanding the training set to include the full dataset. This methodology was devised to simulate the simplest form of a warm-start scenario, wherein the model is incrementally updated with new data batches over time. Our approach mirrors this methodology to emulate a real-world scenario of periodic data arrival, particularly pertinent in medical imaging applications where continuously updating the model is not just beneficial but often necessary for capturing evolving clinical patterns.

Q3: The methodology employed for splitting the datasets into training, validation, and testing categories has been mentioned. Could the authors provide additional details on whether the splits were randomly executed or if a specific stratification process was adopted? I also would like to know, whether the split has been performed as subject-independent or subject-dependent. If it’s not the latter case, I may suggest the author(s) to justify it.

Author response: We are grateful for the reviewer's attention to the important details of data splitting in our study which is a crucial factor that significantly influences model training and validation outcomes. Our division into training, validation, and test sets was executed at the subject level. This approach was deliberately chosen to prevent data leakage and mitigate biased training. Splitting the data at the subject level ensures that images from the same subject do not appear in multiple subsets (training, validation, and testing), thereby preventing the model from gaining an unfair advantage that could artificially inflate performance metrics.

Q4: The author(s) have already reported several works related to DNN for adult and pediatric chest X-ray-based diagnosis. How this present work is differ from the models which are developed in the earlier works? Could you justify it?

Author response: While numerous studies have explored the efficacy of DL models on adult CXRs, literature is limited regarding studying the impact of different model initialization techniques on model performance. Our work fills this gap by examining three distinct initialization strategies: Cold-start, Warm-start, and Shrink and Perturb start, and their impact on model generalizability. Existing literature primarily concentrates on adult populations while overlooking the pediatric demographic. Our study focuses on studying the generalizability of models trained on adult CXRs when applied to pediatric cases, using various model initialization techniques, thereby addressing a significant research gap. Our work simulates real-world challenges by focusing on dynamically arriving data for model training, a scenario commonly encountered in medical imaging but rarely addressed in existing literature. We introduce novel weight-level ensemble techniques, consisting of F-score-weighted Sequential Least-Squares Quadratic Programming (F-SLSQP) and Attention-Guided Ensembles with Learnable Fuzzy Softmax to further improve performance and generalizability. By addressing these multi-faceted challenges, our research contributes an improved understanding of model generalizability across patient populations that extends beyond the scope of existing studies.

Q5: It would be good to include some more potential references in the recent year (especially in 2023) and also try to avoid self-citations if it is not relevant to this present work.

Author response: We are appreciative of the reviewer's insightful recommendation to incorporate recent references and to be cautious with self-citations. We have updated our manuscript to include recent studies from 2023 that are closely aligned with our research themes of model generalizability and ensemble learning.

Regarding the inclusion of self-citations, we wish to clarify that these references are integral to the present work. They provide foundational methodologies and frameworks that have been adapted and extended in this study, particularly in the domains of ensemble learning, model generalizability, and their specific application to chest X-ray analysis.

Author action: The following references are added per the reviewer’s suggestions.

18. Huy VTQ, Lin CM. "An Improved Densenet Deep Neural Network Model for Tuberculosis Detection Using Chest X-Ray Images." IEEE Access. 2023;11: 42839–42849. doi:10.1109/ACCESS.2023.3270774

41. Umapathy S, Murugappan M, Bharathi D, Thakur M. "Automated Computer-Aided Detection and Classification of Intracranial Hemorrhage Using Ensemble Deep Learning Techniques." Diagnostics. 2023;13: 2987. doi:10.3390/diagnostics13182987

47. Mei X, Liu Z, Robson PM, Marinelli B, Huang M, Doshi A, et al. RadImageNet: An Open Radiologic Deep Learning Research Dataset for Effective Transfer Learning. Radiol Artif Intell. 2022;4. doi:10.1148/ryai.210315

Q6: It has been noted that pediatric chest X-rays were partitioned into three distinct groups. Could the rationale behind this stratification be elaborated further with some clinical context?

Author response: We are thankful to the reviewer for posing an insightful question on the rationale behind the stratification of pediatric CXRs into three distinct age groups. Our partitioning was informed by various clinical and developmental considerations as mentioned below:

**Developmental Stages:** We divided the pediatric population into three groups: Group 1 (1 day to <24 months), Group 2 (24 months to <11 years), and Group 3 (11 years to <18 years). This stratification aligns with critical stages in lung and thoracic development, as documented in previous studies (Candemir et al., 2015 [30]).

**Anatomical Variations:** Pediatric thoracic anatomy evolves significantly from infancy to adolescence. For example, the proportions and shapes of the lungs, airways, and thoracic cavity differ substantially, affecting radiographic appearances across these age groups.

**Disease Manifestation:** Pediatric diseases can manifest differently depending on the age group. For instance, certain pathologies that are typically seen in younger children may not be as prevalent in adolescents, and vice versa.

**Imaging Protocols:** Different age groups often necessitate variations in imaging protocols, such as exposure settings, which can result in differences in image intensity and contrast. Furthermore, the level of patient cooperation, which affects inspiration depth, can vary significantly among younger and older pediatric patients.

Our study uniquely addresses the challenge of model generalizability across these various pediatric age groups, each with its own set of clinical and imaging characteristics. By doing so, we contribute to closing the knowledge gap concerning the performance of DL models when applied to both adult and pediatric CXRs across a range of developmental stages.

Q7: The decision to resize the images to a resolution of 256×256 for model training is mentioned. Could the authors shed light on the factors that influenced this choice of resolution?

Author response: We appreciate the reviewer's interest in our choice of image resolution for model training. The decision to resize images to a 256×256 resolution was grounded in both empirical studies and computational considerations. Our choice was influenced by prior work in the field, notably the study by Sabottke et al. [20] which examined CNN performance across varying resolutions for multiple CXR diagnoses. Their findings indicated that maximum AUCs were achieved within the image resolution range of 256×256 to 448×448 pixels for several radiological features. In our previous research titled “Assessing the Impact of Image Resolution on Deep Learning for TB Lesion Segmentation on Frontal Chest X-rays” (Rajaraman et al. [32]), we conducted extensive empirical evaluations to identify the optimal image resolution for Tuberculosis (TB)-consistent lesion segmentation task. Our results demonstrated peak performance metrics, including IoU, Dice, and SSIM, at a 256×256-pixel resolution. At this resolution, the images retain sufficient detail to allow for accurate diagnosis. Also, this resolution strikes a balance between computational efficiency and model performance. Also, several other studies applying DL to CXR analysis opt for resolutions around 256×256, further validating our choice as a standard practice.

Q8: In eqn 1, the author mentioned a constant value of 0.01 for alpha and beta and it has been referred from the earlier works. How do you justify that the values reported in the earlier works also fit your present work?

Author response: We thank the reviewer for these insightful comments. The method proposed in the Shrink and Perturb technique [10] involves shrinking the model weights by scaling the weights with a factor α and incorporating a small noise β to accelerate DL model convergence and enhance generalization. The original authors extensively experimented with different β values and concluded that a value of 0.01 yielded optimal performance, regardless of the weight scaling factor α. Given that this finding was based on comprehensive evaluations, we fixed β=0.01. Unlike β, α is task-dependent and requires careful tuning. To ascertain the most appropriate α value for our specific application, we employed Bayesian optimization via Gaussian Process minimization [37]. We defined a continuous search space for α in the range [0.1, 0.9] and iteratively trained and validated the model to converge to the optimal α value that minimizes validation loss. This adaptability ensures that the model is finely tuned to the characteristics of chest X-ray images, which can vary in terms of contrast, noise, and anatomical structures. The use of Bayesian optimization for hyperparameter tuning ensures that our choice of α is not arbitrary but optimized for the task at hand. By adopting this dual strategy, that is, empirical β selection based on prior research and adaptive α optimization tailored to our specific task, we aim to achieve a balance between leveraging existing scientific knowledge and adapting to the unique requirements of DL-based CXR analysis.

Q9: Why there is no performance comparison between the present work with the relevant works in the literature is not reported?

Author response: We are appreciative of the reviewer's inquiry concerning the absence of a direct performance comparison with existing literature. To the best of our understanding, our study is the first to systematically investigate the impact of multiple model initialization techniques specifically on CXR analysis. Our research scope extends to the cross-population generalizability of models trained on adult CXRs to pediatric populations. This addresses a crucial gap in existing literature and introduces unique methodological and empirical considerations.

Further contributing to the originality of our work are the novel weight-level ensemble techniques we have proposed. These techniques aim to boost both performance and generalizability, thereby adding another layer of novelty that is not present in existing studies.

Given the aforementioned novel aspects, we find that there are no directly comparable studies in the existing literature that address the same set of challenges and research questions. As more studies adopt similar methodologies or tackle similar questions, a more comprehensive performance comparison will become feasible.

Q10: Whether the proposed solution in the present work also satisfy the network which is used for multi-class classification using X-ray images? Will it be suitable for other modality based networks (ex: CT scan, MRI, etc)

Author response: We are grateful to the reviewer for raising this insightful question, which taps into the broader applicability and versatility of our proposed methodologies. While our current work focuses on binary classification, the underlying principles of model initialization and ensemble techniques are fundamentally agnostic to the number of classes. Furthermore, our novel ensemble methods are designed to aggregate knowledge from multiple models, making them inherently adaptable to multi-class scenarios.

Our techniques are expected to be beneficial for other imaging modalities like CT scans and MRIs. These modalities share similar challenges with CXRs, such as the need for robustness, generalizability, and efficient computational performance. The weight initialization and ensemble methods we propose are rooted in general principles of deep learning and are not specific to CXR images, thereby making them adaptable to other modalities.

Q11: The choice of the VGG-16 model, albeit with certain modifications, is intriguing. Could the authors clarify the reasoning behind this particular selection and provide insight into how the performance might have been affected if alternate models were employed?

Author response: We extend our thanks to the reviewer for this perceptive query about our choice of the VGG-16 model architecture. VGG-16 is renowned for its simplicity and effectiveness in various computer vision tasks, including medical image classification. Several previous studies have demonstrated its robust performance in CXR analysis [34–37].

It's crucial to note that the primary focus of our study is not model selection per se, but rather an investigation into the impact of model initialization techniques on generalization. Therefore, we aimed for a model that is widely accepted and validated in the medical imaging community to allow for a focused analysis.

The methodologies we propose for model initialization and ensemble learning are designed to be model-agnostic. This means they can be readily adapted to other architectures, whether they are shallower networks like AlexNet or more complex architectures like ResNets and DenseNets.

While a modified VGG-16 architecture was employed in our study, we acknowledge that the performance could potentially differ if alternate architectures were used. However, given the model-agnostic nature of our initialization and ensemble techniques, we expect that they would also enhance the performance of other models, although the magnitude of the improvement may vary.

Q12: The utilization of Bayesian optimization for selecting the value in the ‘shrink and perturb’ model is highlighted. Could the authors elaborate on the factors that informed this decision?

Author response: We deeply appreciate the reviewer's inquiry into our choice of Bayesian optimization for tuning the weight scaling factor α in the “shrink and perturb” strategy. Previous work by Ash & Adams [10] has primarily used discrete values for α and β. The original authors extensively experimented with different β values and concluded that a value of 0.01 yielded optimal performance, regardless of the weight scaling factor α. Given that this finding was based on comprehensive evaluations, we fixed β=0.01. Unlike β, α is task-dependent and requires careful tuning. We adopt a Bayesian optimization framework via Gaussian Process (GP) minimization [37] to search for the optimal α within a continuous interval, specifically in the range [0.1, 0.9]. This allows for a more nuanced and effective identification of α values that may not be captured in a discrete search space.

Bayesian optimization surpasses traditional methods like grid search or random search by exploring the search space more thoroughly and converging efficiently. Another pivotal advantage of using GP minimization is its ability to reduce susceptibility to local minima, a critical concern in complex medical imaging tasks. By modeling the objective function as a Gaussian process sample, we ensure a global optimization strategy. Our method involves a function that accepts *α* as input. It instantiates and compiles the model with current weights, trains, and validates the model, and stores the best model weights, validation loss, α value, and training history. This is performed for 100 function calls and 30 random starts, aiming to converge to an α that minimizes the validation loss. We believe the successful application of Bayesian optimization opens avenues for its broader utilization in optimizing other facets of DL models for medical imaging tasks.

Q13: The choice of F-score-weighted Sequential Least-Squares Quadratic Programming and Attention-Guided Ensembles with Learnable Fuzzy Softmax, among other existing ensemble methods for aggregating weight parameters, is interesting. Could the authors provide more insights into the rationale behind this choice?

Author response: We highly appreciate the reviewer's interest in our proposed ensemble methods, specifically, the F-score-weighted Sequential Least-Squares Quadratic Programming (F-SLSQP) and Attention-Guided Ensembles with Learnable Fuzzy Softmax (AGELFS). Our decision was guided by several considerations. Traditional ensemble methods often resort to simple or weighted averaging of predictions from multiple models. However, this approach might not capture the intricate relationships between diverse weight initializations and their subsequent learning dynamics. Hence, we shifted our focus to weight-level ensembles, which offer a more nuanced way to capitalize on the collective intelligence of multiple models.

Our proposed weight-level ensembles tap into the complementary strengths of different weight initializations. The F-SLSQP-based weighted ensemble aims to find the optimal multiplication factors for combining model weights, thereby tailoring the ensemble to the specific characteristics of the task at hand. AGELFS leverages attention mechanisms to accentuate the most informative features from each model, thereby providing a more adaptive ensemble. The incorporation of a learnable Fuzzy Softmax layer adds another layer of adaptability by controlling the level of uncertainty in output probabilities.

Our ensemble methods are grounded in mathematical optimization and learning theory. The F-SLSQP method involves a well-defined optimization process, and the AGELFS method builds upon established attention mechanisms and softmax variations, adding a level of scientific rigor to our methodology.

Q14: Additional details on the Clopper-Pearson Exact methodology and Z-test performed for the statistical significance analysis would be appreciated. Could the authors specify the null and alternative hypotheses established for these tests?

Author response: We greatly appreciate the reviewer's request for additional details on our statistical methods, specifically, the Clopper-Pearson Exact methodology and the CI-based Z-test employed for establishing statistical significance. We outline our null and alternate hypothesis as follows: The Null Hypothesis (H_0_) is that there is no statistically significant difference in performance between the different weight initialization models compared, as measured by the Matthews Correlation Coefficient (MCC). The Alternate Hypothesis (H_1_) is that there is a statistically significant difference in performance between the models compared.

We assess model performance using a suite of metrics including balanced accuracy, precision, recall, AUPRC, F-score, and MCC, with a special focus on MCC and recall for statistical significance analysis. We use the Clopper-Pearson Exact method to compute the 95% binomial confidence intervals (CIs) for MCC. This exact method is particularly suitable for medical data, which often has imbalanced or small sample sizes. For each model, we calculate the Standard Error (SE). We compute the difference in MCC and SE between the models being compared. We then calculate the Z-score and the corresponding p-value is obtained via Z-tables. A p-value threshold of 0.05 is employed for determining statistical significance. If the p-value is less than 0.05, we conclude that the difference in performance is statistically significant, thereby rejecting the null hypothesis in favor of the alternative. We apply a similar methodology to evaluate the statistical significance of recall metrics for the proposed weight-level ensembles. By employing this statistical framework, we ensure that our findings are not only compelling but also backed by quantifiable evidence.

Author action: Lines 299 – 315 are modified to include details of the Null and Alternate hypotheses.

Q15: The authors mention observing that models initialized with ImageNet-pretrained weights demonstrated superior generalizability over randomly initialized counterparts, contradicting certain findings for non-medical images. Could the authors share their thoughts on why this difference in results might occur between medical and non-medical images?

Author response: We appreciate the reviewer's query about the contrasting outcomes of model initialization techniques between medical and non-medical image domains. Previous research by Ash & Adams [10] has indicated that warm-start strategies often underperform in terms of generalizability for non-medical images. This could be attributed to the high level of inherent variability in non-medical images, including differing backgrounds, object orientations, and lighting conditions, which might make models initialized with pre-trained weights prone to overfitting specific features irrelevant to the task at hand.

When we transition to the domain of medical images, the landscape changes dramatically due to several unique aspects: Medical images like CXRs are highly homogenous in their appearance. They have their own set of characteristics including contrast, resolution, and noise levels which tend to vary very little across the images. Further difference between normal and abnormal tend to be in intricate variations in the anatomical structures and physiological processes critical for medical diagnostics. Variability in patient positioning, imaging protocols, and the skill level of medical personnel also affect the image quality, which in turn affects the model learning. Medical datasets also often suffer from class imbalance, with far greater “normal” images and significantly fewer instances of pathological conditions. Also, the sensitive nature of medical data imposes additional constraints on data availability and usage. Given these characteristics, pre-trained models may offer an edge as they are trained on images with much greater visual variety and offer a start with a more structured feature space that can be fine-tuned for the specific medical task, thereby facilitating better generalizability. The warm-start strategy, in the context of medical images, can provide a useful initialization that is more aligned with the complex, yet somewhat standardized, features that are often present in medical datasets.

Q16: The reported results indicate that weight-level ensembles of these models achieved significantly higher recall. Could the authors elaborate on how this impacts other performance metrics, such as precision or the F1 score?

Author response: We appreciate the reviewer's interest in understanding the effect of weight-level ensembles on various performance metrics, notably recall, precision, and F1 score. While we observed marginally higher precision for certain ensembles like the F-SLSQP ensemble of the Cold-IF and Warm-IF models, the improvements were not statistically significant. This suggests that while the ensemble is good at identifying true positives, it doesn't necessarily reduce the false positives to a significant extent. Given that recall showed a significant improvement while precision remained relatively stable, the F1 score showed a marginal increase. The Balanced Accuracy and MCC metrics were also observed to be marginally higher but not significantly so. This aligns with our understanding that while ensembles improve recall, the effect on other metrics is more nuanced due to the balancing act between false positives and true positives. In summary, our weight-level ensembles have significantly improved recall, however, the impact on other metrics like precision and F1 score is more subtle, underlining the complex interplay of factors that contribute to model performance.

Q17: Can the authors outline the potential next steps following this research? Are there any other initialization techniques or ensemble methods the authors plan to explore in future studies? Include the potential limitations and possible future work in the discussion section.

Author response: We appreciate the reviewer's interest in the potential future directions and limitations of this research. We acknowledge the limitations of our current approach, including its sensitivity to the quality of the training data, the computational costs associated with weight-level ensembles, and the need for model explainability. In terms of future research, we plan to extend this work in several ways as mentioned in the revised manuscript. By addressing these avenues and limitations, we aim to make substantive contributions to the ongoing efforts to improve the reliability and effectiveness of DL models in healthcare.

Author action: A part of the “Conclusion and future scope” section is modified as follows:

Our study, however, has several limitations: The current study employs the VGG-16 model due to its simplicity and established performance in medical imaging. In future work, we intend to broaden the architectural landscape by incorporating more modern CNN and vision transformer-based architectures. These architectures offer different layers of complexity and specialization that could potentially yield novel insights into the interplay between model initialization and performance. Given the risk of overfitting with improper initialization, incorporating advanced regularization techniques like dropout, L1/L2 regularization, or even Bayesian Neural Networks could offer additional robustness.

Another intriguing direction is to compare the effects of using pre-trained weights from expansive and specialized medical datasets, such as RadImageNet [47], against those initialized with ImageNet. We plan to train models on this dataset from scratch using randomly initialized weights and compare their performance against models initialized with both cold starts and ImageNet pre-trained weights.

While our proposed weight-level ensemble methods demonstrate promising improvements in performance, they also incur a computational overhead that may not be feasible in resource-constrained settings. Future research could focus on optimizing these ensemble techniques for such environments. Utilizing high-end GPUs and cloud-based computing resources can mitigate this limitation to some extent, enabling the deployment of these advanced ensemble techniques in a broader range of settings.

As our models get more complex and effective, understanding their decision-making process becomes crucial. Future work could focus on making these DL models more interpretable to clinicians. While we have employed Bayesian optimization for certain aspects of our model, a more comprehensive AutoML approach could potentially yield even better results.

Extending the scope of initialization techniques to include demographic information like age, sex, and other medical history could pave the way for more personalized DL models in medical imaging. Another exciting avenue is the integration of various medical imaging modalities like MRI, CT scans, and CXRs within a single unified model. This can be particularly useful in complex diagnoses where multiple types of imaging data are often reviewed by physicians. Pursuing these research directions could help improve medical computer vision DL models for reliable healthcare applications.

**Response to Reviewer #2:**

Q1: The research compares various initialization methods for convolutional neural network models and examines their impact on convergence rate, performance, and generalizability when applied to chest x-ray images. Despite the distinction between the ImageNet dataset's natural images and medical image features, the models initialized with ImageNet pre-trained weights showcased better performance compared to those with random initialization. The paper is well-written and easy to follow. The overall experimental setup is well-designed and supports the claims. The investigation exclusively employs the VGG framework. Given that VGG is an overparameterized model, it is prone to overfitting sooner than other models. Delving into more recent architectures like ResNest, DenseNet, ConvNext, and others could offer additional insights. There is an absence of a comparison involving pre-training weights from expansive radiology datasets, such as RadImageNet. Assessing the relative impacts of ImageNet pre-training versus RadImageNet training could be useful. This aspect might be an avenue for further research.

Author response: We extend our sincere gratitude to the reviewer for the insightful comments about our choice of the VGG-16 model architecture and for introducing us to the expansive RadImageNet dataset. VGG-16 is renowned for its simplicity and effectiveness in various computer vision tasks, including medical image classification. Several previous studies have demonstrated its robust performance in CXR analysis [34–36]. The primary focus of our study is not model selection per se, but rather an investigation into the impact of model initialization techniques on generalization. Therefore, we aimed for a model that is widely accepted and validated in the medical imaging community to allow for a focused analysis. However, the methodologies we propose for model initialization and ensemble learning are designed to be model-agnostic. This means they can be readily adapted to other architectures, whether they are shallower networks like AlexNet or more complex architectures like ResNest, DenseNet, ConvNext, and others. While a modified VGG-16 architecture was employed in our study, we acknowledge that the performance could potentially differ if alternate architectures were used. However, given the model-agnostic nature of our initialization and ensemble techniques, we expect that they would also enhance the performance of other models, although the magnitude of the improvement may vary. Thus, our choice of VGG-16 was driven by its proven effectiveness and simplicity, allowing us to concentrate on the novel aspects of model initialization and ensemble learning. Per the reviewer’s comments, we do agree with the fact that employing a variety of architectures like Inception-ResNet-v2, ResNet50, DenseNet121, and InceptionV3, especially those used in the original RadImageNet paper, could provide additional dimensions to our findings. In terms of future research, we plan to train these models from scratch using the RadImageNet dataset and compare their performance against models initialized with both cold starts and ImageNet pre-trained weights. The methodologies we've proposed for model initialization and ensemble learning can be readily adapted to these architectures, and other datasets like RadImageNet. We believe this would be a compelling avenue for future research and we have added this to our future work section, citing the relevant article as recommended.

Author action: A part of the “Conclusion and future scope” section is modified as follows:

Our study, however, has several limitations: The current study employs the VGG-16 model due to its simplicity and established performance in medical imaging. In future work, we intend to broaden the architectural landscape by incorporating more modern CNN and vision transformer-based architectures. These architectures offer different layers of complexity and specialization that could potentially yield novel insights into the interplay between model initialization and performance. Given the risk of overfitting with improper initialization, incorporating advanced regularization techniques like dropout, L1/L2 regularization, or even Bayesian Neural Networks could offer additional robustness.

Another intriguing direction is to compare the effects of using pre-trained weights from expansive and specialized medical datasets, such as RadImageNet [47], against those initialized with ImageNet. We plan to train models on this dataset from scratch using randomly initialized weights and compare their performance against models initialized with both cold starts and ImageNet pre-trained weights.

While our proposed weight-level ensemble methods demonstrate promising improvements in performance, they also incur a computational overhead that may not be feasible in resource-constrained settings. Future research could focus on optimizing these ensemble techniques for such environments. Utilizing high-end GPUs and cloud-based computing resources can mitigate this limitation to some extent, enabling the deployment of these advanced ensemble techniques in a broader range of settings.

As our models get more complex and effective, understanding their decision-making process becomes crucial. Future work could focus on making these DL models more interpretable to clinicians. While we have employed Bayesian optimization for certain aspects of our model, a more comprehensive AutoML approach could potentially yield even better results.

Extending the scope of initialization techniques to include demographic information like age, sex, and other medical history could pave the way for more personalized DL models in medical imaging. Another exciting avenue is the integration of various medical imaging modalities like MRI, CT scans, and CXRs within a single unified model. This can be particularly useful in complex diagnoses where multiple types of imaging data are often reviewed by physicians. Pursuing these research directions could help improve medical computer vision DL models for reliable healthcare applications.
